# Factors that hinder critical thinking and their resolution: Is active learning the key?

**DOI:** 10.12669/pjms.40.6.9490

**Published:** 2024-07

**Authors:** Akiko Takeuchi, Shigeki Jin, Manabu Murakami, Kotaro Matoba

**Affiliations:** 1 Akiko Takeuchi, DDS, PhD. Center for Cause of Death Investigation, Graduate School of Medicine, Hokkaido University, N15W7 Kita-ku, Sapporo, Hokkaido 060-8638, Japan; 2 Shigeki Jin, PhD. Center for Cause of Death Investigation, Graduate School of Medicine, Hokkaido University, N15W7 Kita-ku, Sapporo, Hokkaido 060-8638, Japan; 3 Manabu Murakami, MD, PhD. Center for Medical Education and International Relations, Faculty of Medicine, Hokkaido University, Hokkaido, Japan; 4 Kotaro Matoba, MD, PhD. Center for Cause of Death Investigation, Graduate School of Medicine, Hokkaido University, N15W7 Kita-ku, Sapporo, Hokkaido 060-8638, Japan

Dear Editor,

The importance of critical thinking is undergoing reevaluation in light of recent information overload, rising medical costs, and an increasing emphasis on multidisciplinary teamwork.^1^ In this context, it is vital for medical students to skillfully apply information to unfamiliar problems rather than relying solely on memorization^1^. This emphasis on critical thinking is also evident in Pakistan, where, akin to medical education in Japan, the decision-making process is being reconsidered, and educational research centered on this topic is underway.^2^ Zeb et al.’s study supporting the effectiveness of team-based learning (TBL) in enhancing students’ critical thinking^2^ aligns with our survey’s suggestion of incorporating student-centered active learning.

To explore this further, we engaged 31 Japanese undergraduate students who had studied liberal arts in basic/clinical medicine during their first to third years but had not yet commenced bedside learning. Through 60-minute interviews, we posed open-ended questions about their understanding of critical thinking, its hindrances, and potential solutions. The interview content was anonymized, transcribed, coded, and categorized based on similarity, following which the data underwent conventional content analysis.

## Five key factors emerged:

1) Ambiguity intolerance; 2) Negative impact of peer criticism on the learning atmosphere; 3) Insufficient teaching/learning time; 4) Ambiguity in evaluation criteria; and 5) Lack of experience among learners/instructors (Table-I). As a solution, students proposed curriculum reforms to allocate time for addressing complex, unknown problems devoid of clear answers. They stressed the significance of student-centered active learning (team-based learning). Additionally, our interviews highlighted the importance of 360-degree peer feedback over one-sided evaluation by instructors.

**Table-I:** Factors that hinder critical thinking according to interviewees’ comments.

Factor	Example of interviewee comments
1. Ambiguity intolerance	“Students always end up debating over which answer to a question is better, but there aren’t any classes where we can talk about questions that don’t have clear answers yet, such as, ‘What’s your take on euthanasia?’ ‘There are treatments for both A and B, but we still haven’t figured out the best method for either.’ I really wish we had that chance.”
2. Negative impact of peer criticism on the learning atmosphere	“Critiquing a person’s thoughts and directly critiquing that person are two totally different things, and I don’t think it’s good to bash someone who shares an opinion. There’s a fine line… (omission) If you go too far, you end up nitpicking everything and will always be suspicious of others.”
3. Insufficient teaching/learning time	“I think that according to conventional beliefs, a wiser person is one who finds the answer smoothly without taking too much time. As with tests, having time constraints puts a damper on critical thinking. I believe if we ditch the notion that it’s better to come up with a fixed correct answer quickly, critical thinking will be promoted.”
4. Ambiguity in evaluation criteria	“If teachers assess students’ responses solely in terms of correctness or incorrectness, it implies a flawed approach to thinking, potentially leading to a decline in student’ self-esteem. Instead, I believe it’s preferable to objectively evaluate the logical coherence of the content. This way, a more comprehensive and constructive assessment can be made.”
5. Lack of experience among learners/instructors	“I feel like we’re lacking a proper system for teaching students critical thinking. I believe it’s essential for teachers, not just for students, to engage in ongoing learning. Perhaps some teachers are just being a bit lazy about it. Those who are responsible for teaching critical thinking really need to put in the effort and study ways to effectively impart these skills.”

Our research in Japan was based on a qualitative study on critical thinking barriers conducted in Iran by Kasaraei et al.^3^ Intriguingly, simultaneous quantitative research^2^ on critical thinking was carried out in Pakistan. The traditional education system, emphasizing passive rote memorization, has been criticized for inhibiting critical thinking. Conversely, active learning, especially TBL, has been shown to positively impact critical thinking, consistent with previous research,^1^ emphasizing the importance of the learning environment.

Through this study, we aim to educate medical students and professionals on critical thinking. Future studies, including paramedic and nursing students as in Zeb et al.’s^2^ study, could further explore the impact of active learning in various healthcare disciplines.

## Authors Contributions:

**AT:** 1. research concept, 2. design of the study, 3. literature review, 4. data collection, 5. data analysis, 6. data interpretation, 7. preparation of initial draft, 8. approval of final draft, and 9. guarantor of the manuscript

**SJ:** 1. research concept, 2. design of the study, 3. literature review, 4. data collection, 5. data analysis, 6. data interpretation, 7. revision of initial draft, and 8. approval of final draft

**MM:** 1. research concept, 2. design of the study, 3. literature review, 4. data interpretation, 5. revision of initial draft, 6. approval of final draft, and 7. corresponding author of the manuscript

**KM:** 1. research concept, 2. design of the study, 3. prior literature review, 4. data interpretation, 5. revision of initial draft, and 6. approval of final draft.
